# A-Kinase Interacting Protein 1 Knockdown Restores Chemosensitivity *via* Inactivating PI3K/AKT and β-Catenin Pathways in Anaplastic Thyroid Carcinoma

**DOI:** 10.3389/fonc.2022.854702

**Published:** 2022-07-28

**Authors:** Haiyan Zheng, Qingyuan Lin, Yamin Rao

**Affiliations:** Department of Pathology, Ninth People’s Hospital, Shanghai Jiao Tong University School of Medicine, Shanghai, China

**Keywords:** AKIP1, anaplastic thyroid carcinoma, doxorubicin sensitivity, cell malignant behaviors, PI3K/AKT and β-catenin pathways

## Abstract

**Background:**

A-kinase interacting protein 1 (AKIP1) promotes tumor progression and chemoresistance in several malignancies; meanwhile, it is related to higher tumor size and recurrence risk of papillary thyroid carcinoma, while the role of AKIP1 in anaplastic thyroid carcinoma (ATC) is unclear. The aim of this study is to explore the effect of AKIP1 knockdown on cell malignant behaviors and doxorubicin resistance in ATC.

**Methods:**

AKIP1 knockdown was conducted in ATC cell lines (8505C and CAL-62 cells) by siRNA; then, cell viability, apoptosis, invasion, PI3K/AKT and β-catenin pathways, and doxorubicin sensitivity were detected. Subsequently, doxorubicin-resistant 8505C cells (8505C/Dox) were established. Additionally, AKIP1 was modified in 8505C and 8505C/Dox cells that underwent doxorubicin treatment by siRNA or overexpression plasmid, followed by cellular function and pathway detection.

**Results:**

AKIP1 was elevated in FRO, 8505C, CAL-62, and KHM-5M cells compared to control cells (all *p* < 0.05). Subsequently, AKIP1 knockdown elevated apoptosis, inhibited viability and invasion, and inactivated PI3K/AKT and β-catenin pathways in 8505C and CAL-62 cells (all *p* < 0.05). AKIP1 knockdown decreased relative cell viability in doxorubicin-treated 8505C and CAL-62 cells; then, AKIP1 was elevated in 8505C/Dox cells compared to 8505C cells (all *p* < 0.05). Furthermore, AKIP1 knockdown restored doxorubicin sensitivity (reflected by decreased cell viability and invasion, and increased apoptosis), but inactivated PI3K/AKT and β-catenin pathways in doxorubicin-treated 8505C/Dox cells. However, AKIP1 overexpression presented an opposite effect on these functions and pathways in doxorubicin-treated 8505C cells.

**Conclusion:**

AKIP1 knockdown decreases cell survival and invasion while promoting sensitivity to doxorubicin *via* inactivating PI3K/AKT and β-catenin pathways in ATC.

## Introduction

Anaplastic thyroid carcinoma (ATC), a rare but the most malignant type of thyroid carcinomas, is characterized by poor differentiation, a highly aggressive nature, and quick metastasis (1–4). Currently, treatments of ATC typically consist of surgery, chemotherapy, radiotherapy, molecular targeted therapy, etc. (1, 5, 6). However, these comprehensive treatments of ATC still fail to demonstrate an obvious survival benefit for ATC patients due to its intrinsic characteristics; thus, ATC is viewed as a devastating disease with a high mortality rate (5). Hence, exploring the potential molecular mechanism and possible target to improve treatment of ATC is crucial and urgent.

A-kinase interacting protein 1 (AKIP1), originally named breast cancer-associated protein 3, facilitates the nuclear translocation of catalytic subunit of protein kinase A (7, 8). Recently, it has been reported that AKIP1 is viewed as a tumor promoter (7–11). For instance, it is proposed that AKIP1 induces the nuclear factor kappa-B (NF-κB)-dependent chemokines to promote angiogenesis and tumor growth in cervical cancer (8). Moreover, AKIP1 elevates vascular endothelial growth factor-C (VEGF-C) to accelerate angiogenesis and lymphangiogenesis in human esophageal squamous cell carcinoma (9). Another interesting study discloses that AKIP1 activates Zinc Finger E-Box Binding Homeobox 1 (ZEB1) to facilitate tumor metastasis in non-small cell lung cancer (10). In addition, AKIP1 is also considered as a regulator of treatment resistance in malignancies (11, 12). For example, AKIP1 upregulates C-X-C motif chemokine ligand (CXCL)1 and CXCL8 to decrease chemoradiation sensitivity in glioblastoma (12). Additionally, AKIP1 interacts with Tap73 to modulate the radiotherapy sensitivity of cervical cancer cells (11). Importantly, recent research has presented that AKIP1 is correlated with advanced tumor features and higher recurrence risk in papillary thyroid carcinoma (13). According to the above-mentioned information, we deduce that AKIP1 may play an important role in tumor progression and treatment resistance in ATC, while related data are scarce.

In the current study, AKIP1 modification was conducted in ATC cell lines, followed by detection of cellular functions, chemotherapy resistance, and downstream pathways, aiming to explore the potential of AKIP1 as a treatment target for ATC. The current study discovered that AKIP1 knockdown inhibited cell viability and invasion but promoted cell apoptosis, as well as restored doxorubicin sensitivity *via* inactivating PI3K/AKT and β-catenin pathways in ATC, indicating that targeting AKIP1 might provide a new treatment choice for ATC.

## Methods

### Cell Lines

Human normal thyroid cell line (Nthy-ori 3-1) was purchased from the European Collection of Authenticated Cell Cultures (ECACC). Human ATC cell lines, including FRO, 8505C, C643, CAL-62, and KHM-5M, were purchased from the National Collection of Authenticated Cell Cultures (Shanghai, China). Cells were maintained in DMEM (Lonza, Swiss) (8505C and CAL-62) or RPMI-1640 medium (Lonza, Swiss) (Nthy-ori 3-1, FRO, C643, and KHM-5M) containing 10% fetal bovine serum (FBS) (Sigma, USA) and 1% penicillin/streptomycin (Beyotime, China) at 37°C in an incubator with 5% CO_2_. The doxorubicin-resistant 8505C cells (8505C/Dox) were established from the parental 8505C cells by exposing cells to gradually increasing concentrations of doxorubicin (Sigma, USA) from 0.1 μM to 10 μM over 8 months (14). 8505C/Dox cells were cultured in culture medium containing 2 μM doxorubicin to maintain drug resistance phenotype.

### Cell Transfection

The AKIP1 siRNA (si-AKIP1) and negative control (si-NC) were commercially designed and synthesized by Shanghai GenePharma Co., Ltd. (Shanghai, China). The AKIP1 overexpression plasmids (pcDNA-AKIP1) and negative control (pcDNA-NC) were obtained from Guangzhou Ribobio Co., Ltd. (Guangzhou, China). ATC cells were cultured and transfected with 50 nM siRNA (si-AKIP1 or si-NC) or 0.8 μg of plasmids (pcDNA-AKIP1 or pcDNA-NC) with Lipofectamine^®^ 3000 (Invitrogen, USA) for 6 h. The AKIP1 siRNA sequence was as follows: sense, 5’ GTGGGCTCAAATGACTTAATT 3’; antisense, 5’ TTAAGTCATTTGAGCCCACTT 3’. The non-transfected cells served as normal controls.

### Drug Sensitivity Assay

The 8505C cells were incubated with 0, 0.2, 0.4, 0.8, 1.6, 3.2, and 6.4 μM doxorubicin; CAL-62 cells were incubated with 0, 0.02, 0.04, 0.08, 0.16, 0.32, and 0.64 μM doxorubicin; and 8505C/Dox cells were incubated with 0, 2, 4, 8, 16, 32, and 64 μM doxorubicin (15, 16). After 48 h of treatment, CCK-8 assay was performed by the methods mentioned in the *Cell Viability Assay* subsection and half maximal inhibitory concentration (IC_50_) was calculated using the sigmoidal dose–response function of the GraphPad Prism software (Version 7.0) (17).

### Doxorubicin Treatment

To assess whether AKIP1 participated in the drug resistance of ATC cells, the 8505C cells were transfected with pcDNA-AKIP1 and cultured with 1.6 μM doxorubicin (selected by IC_50_); meanwhile, 8505C/Dox cells were transfected with si-AKIP1 and cultured with 16 μM doxorubicin. Briefly, 8505C cells were divided into five groups, including the Normal group (non-treated), the pcDNA-NC group (transfected with NC plasmids), the pcDNA-AKIP1 group (transfected with AKIP1 overexpression plasmids), the pcDNA-NC and Dox group (transfected with NC plasmids and treated with 1.6 μM doxorubicin), and the pcDNA-AKIP1 and Dox group (transfected with AKIP1 overexpression plasmids and treated with 1.6 μM doxorubicin). Analogously, 8505C/Dox cells were divided into five groups, including the Normal group (non-treated), the si-NC group (transfected with NC siRNA), the si-AKIP1 group (transfected with AKIP1 siRNA), the si-NC and Dox group (transfected with NC siRNA and treated with 16 μM doxorubicin), and the si-AKIP1 and Dox group (transfected with AKIP1 siRNA and treated with 16 μM doxorubicin). After 48 h of treatment, further assays were carried out.

### Reverse Transcription Quantitative Polymerase Chain Reaction for AKIP1 Expression

In brief, total RNA from ATC cells was extracted *via* Beyozol (Beyotime, China). Reverse transcription of RNA was completed by the GeneAce Reverse Transcriptase Kit (Nippon, Japan). The PCR program was implemented using the SYBR^®^ Premix DimmerEraser™ kit (Takara, Japan). The thermal cycle of qPCR was as follows: 1 cycle, 95°C for 30 s; 40 cycles, 95°C for 5 s and 61°C for 30 s. The AKIP1 mRNA expression was evaluated based on the 2^−ΔΔCt^ method with GAPDH as an endogenous control. The primer sequences were listed as follows (5’→3’): AKIP1 forward, CATGGACAACTGTTTGGCGG, AKIP1 reverse: TAGAGCCAGCCTTGCTGAAC; GAPDH forward, GAGTCCACTGGCGTCTTCAC, GAPDH reverse, ATCTTGAGGCTGTTGTCATACTTCT.

### Western Blotting

Western blotting assays of ATC cells were implemented at 48 h after treatment. First of all, ATC cells were lysed by RIPA comprising 1 mM PMSF (Selleck, USA) and the concentration of total proteins was quantified using the bicinchoninic acid kit (Beyotime, China). Secondly, 40 μg of proteins was separated by SDS-PAGE and transferred onto a nitrocellulose membrane (Pall, USA). Then, membranes were blocked using 5% nonfat milk (Beyotime, China), hatched with primary antibodies overnight at 4°C, and subsequently incubated with secondary antibody (1:10,000) (Affinity, China) for 1 h at 37°C. Finally, blots were detected *via* ECL-PLUS kit (Beyotime, China) and analyzed using ImageJ software (version 1.8.0, NIH). The primary antibodies used in this study were bought from Abcam (Cambridge, USA) and listed as follows: anti-AKIP1 (1:500, ab135996), anti-p-PI3K (1:1,000, ab182651), anti-PI3K (1:1,500, ab86714), anti-p-AKT^Ser473^ (1:1,000, ab81283), anti-AKT (1:1,500, ab8805), anti-β-catenin (1:1,000, ab68813), anti-GAPDH (1:5,000, ab181602), and anti-Histone H3 (1:5,000, ab1791).

### Cell Viability Assay

Cell viability was evaluated by the Cell Counting Kit-8 (CCK-8) (Sigma, USA) assay. In brief, ATC cells were plated in 96‐well plates (2 × 10^3^ cells/well) and cultured for 48 h and 72 h. Then, 10 μl of CCK-8 reagent was added and further cultured for another 2 h at 37°C. Finally, the optical density (OD) values at 450 nm were assessed with a microplate reader (Molecular Devices, USA).

### Cell Apoptosis Assay

Cell apoptosis rate was detected using the TUNEL apoptosis kit (Sangon, China). Briefly, at 48 h and 72 h after treatment, ATC cells (1 × 10^4^ cells/well) were fixed with 4% paraformaldehyde (Sangon, China) and permeabilized with 0.1% Triton X-100 (Sangon, China). Then, cells were incubated with TUNEL reagent for 20 min and DAPI solution (Sigma, USA) for 10 min at room temperature (RT), successively. The apoptotic cells were viewed and imaged with a microscope (Olympus, Japan).

### Cell Invasion Assay

At 48 h after treatment, the invasion ability of ATC cells was assessed by the transwell assay. In brief, cells were adjusted to 5 × 10^4^ cells/well with serum-free medium and plated into the upper chamber of Matrigel-coated transwell chamber plates (Life, USA). The lower chambers were covered with 600 μl of DMEM containing 10% FBS. At 24 h after culture, the invasive cells were counted under a microscope after being stained with crystal violet (Beyotime, China) for 10 min at RT (16, 18).

### Statistical Analysis

The experiment was performed in triplicate. One-way ANOVA with Tukey’s or Dunnett’s post-hoc test and Student’s *t*-test were used for comparisons. All statistical analyses were calculated by GraphPad Prism. *p* < 0.05 was considered to be statistically significant.

## Results

### AKIP1 Expression

**Figure 1:**
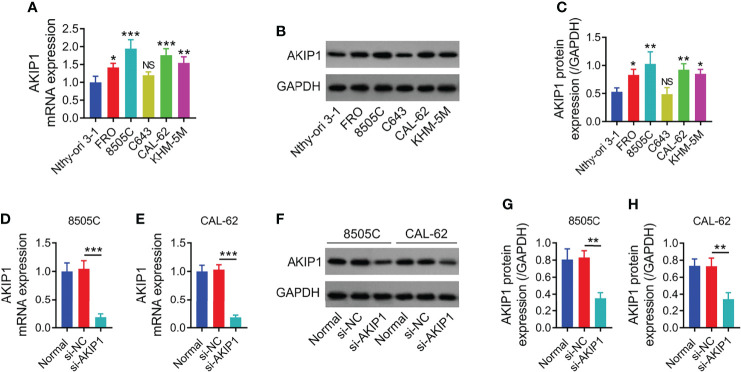
Detection of AKIP1 expression. Comparison of AKIP1 mRNA expression between human normal thyroid cell line and ATC cell lines **(A)**; example image of AKIP1 protein in human normal thyroid cell line and ATC cell lines through Western blot **(B)**; comparison of AKIP1 protein expression between human normal thyroid cell line and ATC cell lines **(C)**; comparison of AKIP1 mRNA expression among groups in 8505C cells **(D)** and CAL-62 cells **(E)** after transfection; detection of AKIP1 protein expression by Western blot in 8505C cells and CAL-62 cells after transfection **(F)**; comparison of AKIP1 protein expression among groups in 8505C cells **(G)** and CAL-62 cells **(H)** after transfection by one-way ANOVA followed by Dunnett’s post-hoc test. ATC, anaplastic thyroid carcinoma; AKIP1, A-kinase interacting protein 1; NS, not significant; *p < 0.05; **p < 0.01; ***p < 0.001.

No difference was found in AKIP1 between Nthy-ori3-1 cells and C643 cells (*p* > 0.05), while AKIP1 was increased in FRO, 8505C, CAL-62, and KHM-5M cells compared to Nthy-ori 3-1 cells (all *p* < 0.05); since AKIP1 was dramatically elevated in 8505C and CAL-62 cells, these two cell lines were selected for further experiments (**Figures 1A–C**). Moreover, 8505C and CAL-62 cells were transfected with AKIP1 siRNA or NC siRNA; data showed that AKIP1 was decreased in 8505C and CAL-62 cells transfected with AKIP1 siRNA compared to those transfected with NC siRNA, suggesting successful transfection (**Figures 1D–H**).

### AKIP1 Knockdown Decreased Cell Survival and Invasion in ATC Cell Lines

**Figure 2:**
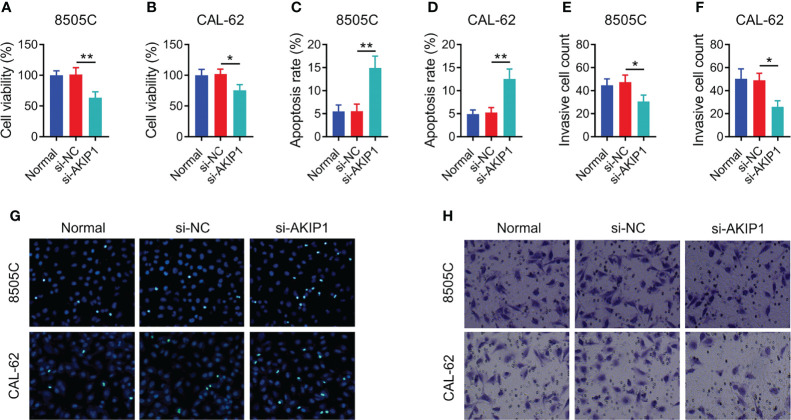
Cellular functions in ATC cell lines at 48 h after siRNA transfection. Comparison of relative cell viability among groups in 8505C cells **(A)** and CAL-62 cells **(B)** after transfection; comparison of apoptosis among groups in 8505C cells **(C)** and CAL-62 cells **(D)** after transfection; comparison of invasive cell count among groups in 8505C cells **(E)** and CAL-62 cells **(F)** after transfection by one-way ANOVA followed by Dunnett’s post-hoc test; example image of cell apoptosis through TUNEL Apoptosis Assay Kit **(G)** and example image of cell invasion through transwell assay **(H)** in 8505C cells and CAL-62 cells after transfection. ATC, anaplastic thyroid carcinoma; siRNA, small interfering RNA; AKIP1, A-kinase interacting protein 1; *p < 0.05; **p < 0.01.

In order to explore the regulation of AKIP1 on cellular functions, cell viability, apoptosis, and invasion assays were conducted. The data showed that AKIP1 knockdown elevated apoptosis but declined cell viability and invasive cell count in both 8505C and CAL-62 cells at 48 h after transfection (all *p* < 0.05); moreover, the rates of inhibition of survival of 8505C and CAL-62 cells transfected with si-AKIP1 were 37.14% and 25.82%, respectively, at 48 h after transfection; meanwhile, the apoptosis rate of normal 8505C cells, and 8505C cells transfected with si-NC and si-AKIP1 was 5.52%, 5.54%, and 14.93%, respectively; moreover, the apoptosis rate of normal CAL-62 cells, and CAL-62 cells transfected with si-NC and si-AKIP1 was 4.92%, 5.26%, and 12.52%, respectively (**Figures 2A–H**). Furthermore, cell viability and apoptosis presented similar trends at 72 h after AKIP1 knockdown (**Supplementary Figures 1A–E**).

### AKIP1 Knockdown Suppressed PI3K/AKT and β-Catenin Pathways in ATC Cell Lines

**Figure 3:**
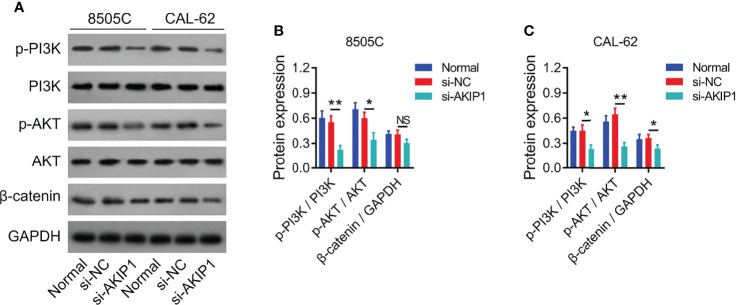
PI3K/AKT and β-catenin pathways in ATC cell lines after siRNA transfection. Detection of p-PI3K, PI3K, p-AKT, AKT, and β-catenin protein expressions by Western blot in 8505C cells and CAL-62 cells after transfection **(A)**; comparison of p-PI3K/PI3K, p-AKT/AKT, and β-catenin expressions among groups in 8505C cells **(B)** and CAL-62 cells **(C)** after transfection by one-way ANOVA followed by Dunnett’s post-hoc test. ATC, anaplastic thyroid carcinoma; siRNA, small interfering RNA; AKIP1, A-kinase interacting protein 1; NS, not significant; PI3K, phosphatidylinositol-3-kinase; AKT, protein kinase B; GAPDH, glyceraldehyde-3-phosphate dehydrogenase; *p < 0.05; **p < 0.01.

Previous studies have presented that AKIP1 modulates PI3K/AKT and β-catenin pathways to regulate tumor progression (12, 19, 20); thus, the effect of AKIP1 knockdown on these pathways in ATC cell lines was explored by Western blot, which revealed that AKIP1 knockdown inhibited the phosphorylation of PI3K and AKT in 8505C and CAL-62 cells (both *p* < 0.05); meanwhile, AKIP1 knockdown inhibited expression of β-catenin in CAL-62 cells (*p* < 0.05) but not in 8505C cells (*p* > 0.05) (**Figures 3A–C**). Furthermore, AKIP1 knockdown inhibited the nuclear translocation of β-catenin in 8505C and CAL-62 cells (both *p* < 0.01) (**Supplementary Figures 2A–C**).

### AKIP1 Was Elevated in 8505C/Dox Cells

**Figure 4:**
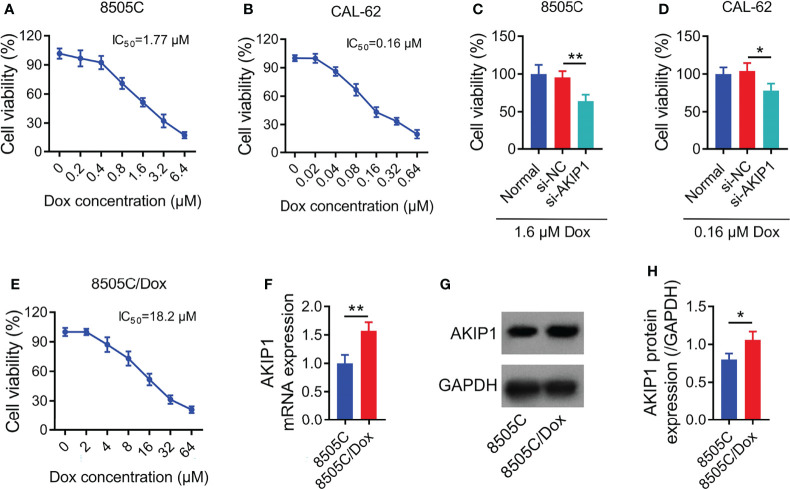
AKIP1 involved in doxorubicin sensitivity in ATC cell lines. Relative cell viability of 8505C cells **(A)** and CAL-62 cells **(B)** under different concentrations of doxorubicin treatment without siRNA transfection; comparison of relative cell viability between groups in 8505C cells after siRNA transfection under 1.6 μM doxorubicin treatment **(C)** and CAL-62 cells after siRNA transfection under 0.16 μM doxorubicin treatment **(D)**; relative cell viability under different concentrations of doxorubicin treatment in 8505C/Dox cells without siRNA transfection **(E)**; comparison of AKIP1 mRNA expression between 8505C cells and 8505C/Dox cells without siRNA transfection or doxorubicin treatment **(F)**; detection of AKIP1 protein expression by Western blot in 8505C cells and 8505C/Dox cells without siRNA transfection or doxorubicin treatment **(G)**; comparison of AKIP1 protein expression between 8505C cells and 8505C/Dox cells without siRNA transfection or doxorubicin treatment **(H)** by one-way ANOVA followed by Dunnett’s post-hoc test and Student’s *t*-test. ATC, anaplastic thyroid carcinoma; AKIP1, A-kinase interacting protein 1; Dox, doxorubicin; IC_50_, half maximal inhibitory concentration; *p < 0.05; **p < 0.01.

Relative cell viability was declined along with the increasing concentration of doxorubicin in 8505C and CAL-62 cells; meanwhile, the IC_50_ concentration of doxorubicin was identified as 1.77 μM in 8505C cells and 0.16 μM in CAL-62 cells (**Figures 4A, B**). Furthermore, 1.6 μM doxorubicin was used to treat 8505C cells and 0.16 μM doxorubicin was used to treat CAL-62 cells after transfection; data showed that AKIP1 knockdown decreased relative cell viability in 8505C cells and CAL-62 cells under doxorubicin treatment (both *p* < 0.05) (**Figures 4C, D**). In addition, AKIP1 knockdown declined relative cell viability in 0.8 μM, 1.6 μM, and 3.2 μM doxorubicin-treated 8505C cells; meanwhile, AKIP1 knockdown decreased relative cell viability in 0.04 μM, 0.08 μM, 0.16 μM, and 0.32 μM doxorubicin-treated CAL-62 cells (all *p* < 0.05) (**Supplementary Figures 3A, B**). To explore whether AKIP1 participated in drug resistance of ATC, 8505C/Dox cells with an IC_50_ concentration of 18.2 μM were established (**Figure 4E**). Furthermore, AKIP1 was elevated in 8505C/Dox cells compared to 8505C cells (both *p* < 0.05) (**Figures 4F–H**).

### AKIP1 Knockdown Enhanced Doxorubicin Sensitivity, and Inactivated PI3K/AKT and β-Catenin Pathways in 8505C/Dox Cells

**Figure 5:**
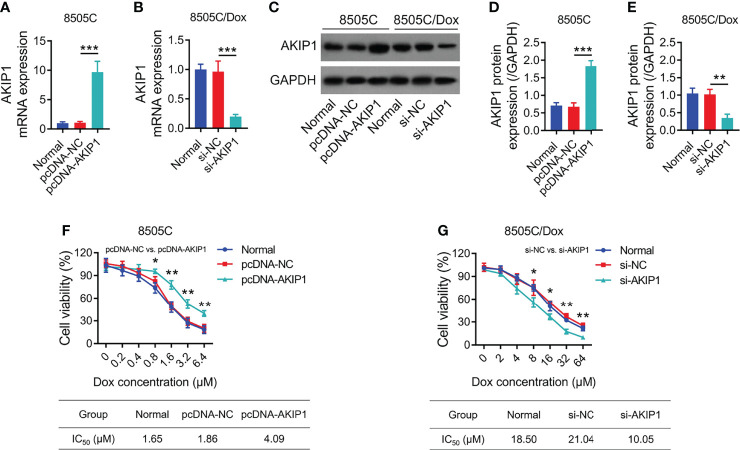
AKIP1 modification regulated ATC cell viability under different concentrations of doxorubicin. Comparison of AKIP1 mRNA expression among groups in 8505C cells **(A)** and 8505C/Dox **(B)** after AKIP1 modification; detection of AKIP1 protein expression by Western blot in 8505C cells and 8505C/Dox after AKIP1 modification **(C)**; comparison of AKIP1 protein expression among groups in 8505C cells **(D)** and 8505C/Dox **(E)** after AKIP1 modification; comparison of relative cell viability among groups in 8505C cells after AKIP1 modification under 0–6.4 μM doxorubicin treatment **(F)** and 8505C/Dox after AKIP1 modification under 0–64 μM doxorubicin treatment **(G)** by one-way ANOVA followed by Dunnett’s post-hoc test. ATC, anaplastic thyroid carcinoma; AKIP1, A-kinase interacting protein 1; Dox, doxorubicin; IC_50_, half maximal inhibitory concentration; siRNA, small interfering RNA; NC, negative control *p < 0.05; **p < 0.01; ***p < 0.001.

To further explore the effect of AKIP1 on drug resistance in ATC cell lines, 8505C cells were transfected with AKIP1 or NC overexpression plasmids; meanwhile, 8505C/Dox cells were transfected with AKIP1 siRNA or NC siRNA; data showed that AKIP1 was elevated in 8505C cells transfected with the AKIP1 overexpression plasmid compared to those transfected with the NC overexpression plasmid (both *p* < 0.001), while AKIP1 was declined in 8505C/Dox cells transfected with AKIP1 siRNA compared to NC siRNA (both *p* < 0.01) (**Figures 5A–E**). Moreover, AKIP1 overexpression elevated relative cell viability in 0.8 μM, 1.6 μM, 3.2 μM, and 6.4 μM doxorubicin-treated 8505C cells; moreover, AKIP1 knockdown decreased relative cell viability in 8 μM, 16 μM, 32 μM, and 64 μM doxorubicin-treated 8505C/Dox cells, as well as promoted the doxorubicin sensitivity in 8505C/Dox cells (IC_50_ in normal cells: 18.50 μm, IC_50_ in siRNA-AKIP1 cells: 10.05 μm) (**Figures 5F, G**).

**Figure 6:**
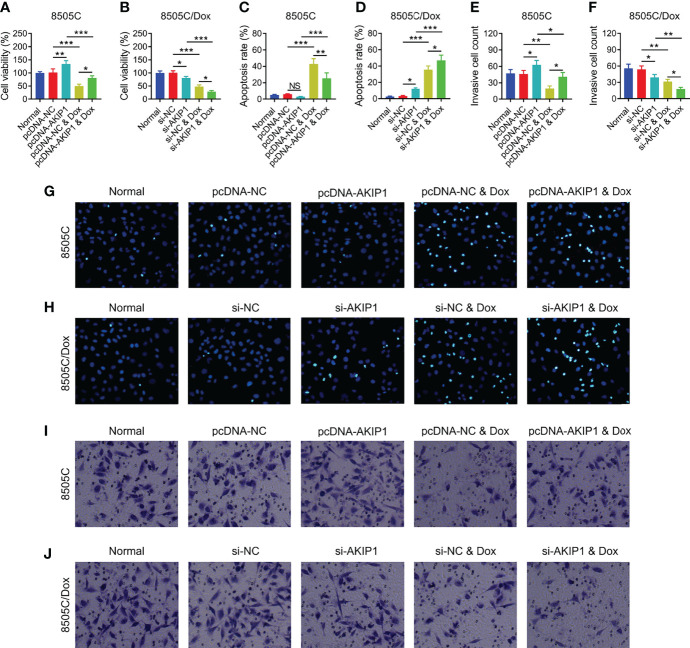
ATC cellular functions after AKIP1 modification and doxorubicin treatment. Comparison of relative cell viability among groups in 8505C cells **(A)** and 8505C/Dox cells **(B)** after AKIP1 modification and doxorubicin treatment; comparison of apoptosis rate among groups in 8505C cells **(C)** and 8505C/Dox cells **(D)** after AKIP1 modification and doxorubicin treatment; comparison of invasive cell count among groups in 8505C cells **(E)** and 8505C/Dox cells **(F)** after AKIP1 modification and doxorubicin treatment by one-way ANOVA followed by Tukey’s post-hoc test; example image of cell apoptosis through TUNEL Apoptosis Assay Kit in 8505C cells **(G)** and 8505C/Dox cells **(H)** after AKIP1 modification and doxorubicin treatment; example image of cell invasion through transwell assay in 8505C cells **(I)** and 8505C/Dox cells **(J)** after AKIP1 modification and doxorubicin treatment. ATC, anaplastic thyroid carcinoma; AKIP1, A-kinase interacting protein 1; Dox, doxorubicin; NC, negative control; siRNA, small interfering RNA; TUNEL, terminal deoxynucleotidyl transferase-mediated dUTP-biotin nick end labeling; NS, not significant; *p < 0.05; **p < 0.01; ***p < 0.001.

**Figure 7:**
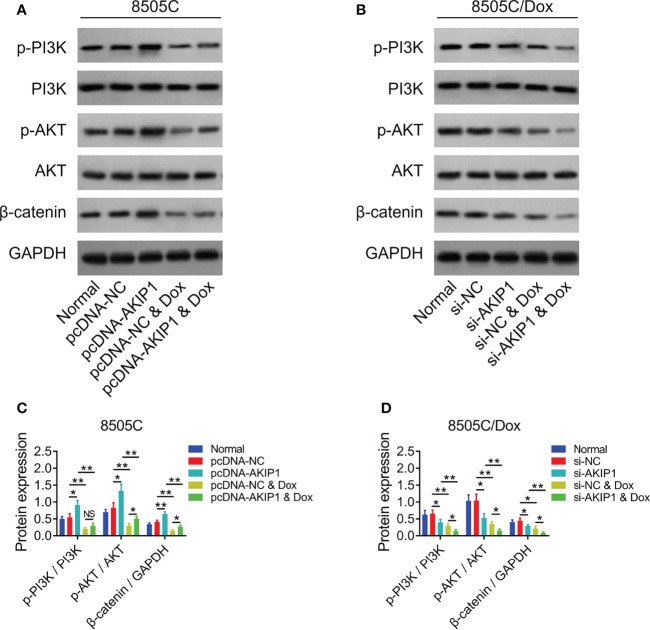
PI3K/AKT and β-catenin pathways after AKIP1 modification and doxorubicin treatment in ATC cell lines. Detection of p-PI3K, PI3K, p-AKT, AKT, and β-catenin protein expressions by Western blot in 8505C cells **(A)** and 8505C/Dox cells **(B)** after AKIP1 modification and doxorubicin treatment; comparison of p-PI3K/PI3K, p-AKT/AKT, and β-catenin expressions among groups in 8505C cells **(C)** and 8505C/Dox cells **(D)** after AKIP1 modification and doxorubicin treatment by one-way ANOVA followed by Tukey’s post-hoc test. ATC, anaplastic thyroid carcinoma; AKIP1, A-kinase interacting protein 1; Dox, doxorubicin; NS, not significant; NC, negative control; siRNA, small interfering RNA; PI3K, phosphatidylinositol-3-kinase; AKT, protein kinase B; GAPDH, glyceraldehyde-3-phosphate dehydrogenase; *, p < 0.05; **, p < 0.01.

To further verify the effect of AKIP1 on drug resistance in ATC cell lines, 8505C cells after transfection were treated by 1.6 μM doxorubicin and 8505C/Dox cells after transfection were treated by 16 μM doxorubicin. Data presented that AKIP1 overexpression elevated relative cell viability and invasive cell count, but decreased apoptosis rate in doxorubicin-treated 8505C cells (all *p* < 0.05); furthermore, AKIP1 knockdown decreased relative cell viability and invasive cell count, but elevated apoptosis rate in doxorubicin-treated 8505C/Dox cells (all *p* < 0.05) (**Figures 6A–J**). Moreover, AKIP1 overexpression increased p-AKT and β-catenin in doxorubicin-treated 8505C cells, while AKIP1 knockdown decreased p-PI3K, p-AKT, and β-catenin in doxorubicin-treated 8505C/Dox cells (all *p* < 0.05) (**Figures 7A–D**). In addition, AKIP1 overexpression increased the nuclear translocation of β-catenin in doxorubicin-treated 8505C cells, while AKIP1 knockdown declined the nuclear translocation of β-catenin in doxorubicin-treated 8505C/Dox cells (both *p* < 0.05) (**Supplementary Figures 4A–C**).

## Discussion

ATC accounts for only 2% of all thyroid carcinomas; although it is rare, ATC is viewed as one of the invariably lethal diseases worldwide (1–3). Until now, the prognosis of ATC is relatively poor due to the characteristics of ATC, such as aggressive nature and metastasis at an early stage (4–6). Moreover, partly because ATC lacks thyroid-like phenotype and function, the treatment option of ATC is very limited; meanwhile, ATC usually presents an unfavorable response rate to chemotherapy and other available therapies (21). Given that ATC is a worldwide threat and extremely hard to manage, exploring the potential target to improve treatment is crucial. In the current study, we explored the interaction of AKIP1 with chemosensitivity and pathways in ATC. Interestingly, we discovered that AKIP1 knockdown could inhibit ATC cell survival and invasion while enhancing doxorubicin sensitivity *via* inhibiting PI3K/AKT and β-catenin pathways.

Over the past decades, AKIP1 has drawn a lot of attention in oncology research, due to its important biological role in multiple malignancies (8, 19, 22, 23). For instance, AKIP1 promotes breast cancer cell motility *via* suppressing Akt/glycogen synthase kinase3β/Snail pathways (22); moreover, AKIP1 elevates hepatocellular carcinoma (HCC) metastasis through Wnt/β-catenin/cyclic AMP response element-binding protein pathways (19); furthermore, AKIP1 facilitates gastric cancer metastasis and cell growth and through activating epithelial–mesenchymal transition (23); additionally, AKIP1 accelerates glioblastoma viability, mobility, and chemoradiation resistance *via* the NF-κB pathway (12); meanwhile, AKIP1 is also considered as a molecular determinant of protein kinase in the NF-κB pathway (24); furthermore, AKIP1 is able to bind the p65 subunit of NF-κB and modulate its transcriptional activity (25). Thus, combined with previous studies, we hypothesize that AKIP1 also takes part in the pathophysiology of ATC, while the information about this issue is scarce. In the current study, we disclosed that AKIP1 was elevated in ATC cell lines compared to the human normal thyroid cell line; meanwhile, we also found that AKIP1 knockdown elevated apoptosis but inhibited relative cell viability and cell invasion in ATC cell lines, which was consistent with the results of previous research about other malignancies (8, 19, 22, 23). The possible explanations might be that (1) AKIP1 knockdown could inhibit ATC cell survival and invasion through several approaches (such as inactivating slug-induced epithelial–mesenchymal transition and downregulating CXC-chemokines) (8, 23) and (2) AKIP1 knockdown might decrease ATC cell survival and invasion through modulating PI3K/AKT and β-catenin pathways (results of our subsequent research).

The PI3K/AKT pathway is viewed as a vital modulator among numerous cancers, such as breast cancer and lung adenocarcinoma (26–30). Importantly, a variety of previous studies suggest that the PI3K/AKT pathway also plays an important role in tumor progression and drug resistance in ATC (31–33). For instance, inhibition of the PI3K/AKT pathway suppresses ATC aggressiveness, such as decreasing cell invasion and survival (31–34); meanwhile, inactivating the PI3K/AKT pathway elevates chemosensitivity in ATC (35). In addition, β-catenin dysregulation is usually found in different malignancies, including ATC (32, 36, 37). For instance, β-catenin inactivation declined cell survival and invasiveness in ATC (31, 36, 38); moreover, β-catenin inactivation is able to elevate iodine uptake in ATC (36). In addition, AKIP1 is reported to regulate PI3K/AKT and β-catenin pathways in several malignancies (12, 19, 20). Inspired by previous data, in the current study, we explored the interaction of AKIP1 with PI3K/AKT and β-catenin pathways in ATC, and data showed that AKIP1 knockdown inactivated PI3K/AKT and β-catenin pathways in ATC cell lines.

It is a big challenge for chemoresistance to elevate outcome among ATC patients, which is regulated by several pathways, including PI3K/AKT and β-catenin pathways (6, 21, 26, 36). Moreover, AKIP1 is proposed to modulate chemoresistance in several cancers (11, 12). Thus, we hypothesized that AKIP1 could regulate chemosensitivity through PI3K/AKT and β-catenin pathways in ATC. In the current research, we chose doxorubicin in the chemosensitivity assay, because doxorubicin is the main cornerstone recommended by the National Comprehensive Cancer Network guideline for the treatment of ATC (39, 40). Surprisingly, we discovered that AKIP1 knockdown elevated apoptosis rate, decreased relative cell viability and invasive cell count, and inactivated PI3K/AKT and β-catenin pathways in chemo-resistant ATC cells under doxorubicin treatment, indicating that AKIP1 knockdown restored doxorubicin sensitivity *via* inhibiting PI3K/AKT and β-catenin pathways in ATC. The possible reasons might be that (1) AKIP1 knockdown could decrease excretion of C-X-C motif chemokine ligand families, which consequently elevated chemosensitivity (19, 20, 31) and (2) AKIP1 knockdown could inactivate PI3K/AKT and β-catenin pathways to decline stemness, which indirectly elevated chemosensitivity (41–43).

To be conclusive, AKIP1 knockdown decreases cell survival and invasion, while restoring doxorubicin sensitivity through inactivating PI3K/AKT and β-catenin pathways in ATC, indicating that AKIP1 may be a potential target to improve the treatment of ATC.

## Data Availability Statement

The datasets presented in this study can be found in online repositories. The names of the repository/repositories and accession number(s) can be found in the article/**Supplementary Material**.

## Author Contributions

HZ and QL designed the research study. HZ, QL, and YR performed the research. YR provided help and advice on administration. HZ and QL analyzed the data. HZ, QL, and YR wrote the manuscript. All authors contributed to editorial changes in the manuscript. All authors read and approved the final manuscript.

## Funding

This work was supported by the Project of Biobank (No.YBKB201911) from Shanghai Ninth People’s Hospital, Shanghai Jiao Tong University School of Medicine and the Cross Disciplinary Research Projects of Ninth People's Hospital, Shanghai Jiao Tong University School of Medicine (JYJC202106).

## Conflict of Interest

The authors declare that the research was conducted in the absence of any commercial or financial relationships that could be construed as a potential conflict of interest.

## Publisher’s Note

All claims expressed in this article are solely those of the authors and do not necessarily represent those of their affiliated organizations, or those of the publisher, the editors and the reviewers. Any product that may be evaluated in this article, or claim that may be made by its manufacturer, is not guaranteed or endorsed by the publisher.
