# Supplementary material for: A-Kinase Interacting Protein 1 Knockdown Restores Chemosensitivity via Inactivating PI3K/AKT and β-Catenin Pathways in Anaplastic Thyroid Carcinoma
Source: Front Oncol. 2022 Jul 28;12:854702. doi: 10.3389/fonc.2022.854702 (PMC9366429; doi:10.3389/fonc.2022.854702)
Supplement: Supplementary file 1 [file DataSheet_1.pdf]

Supplementary Material

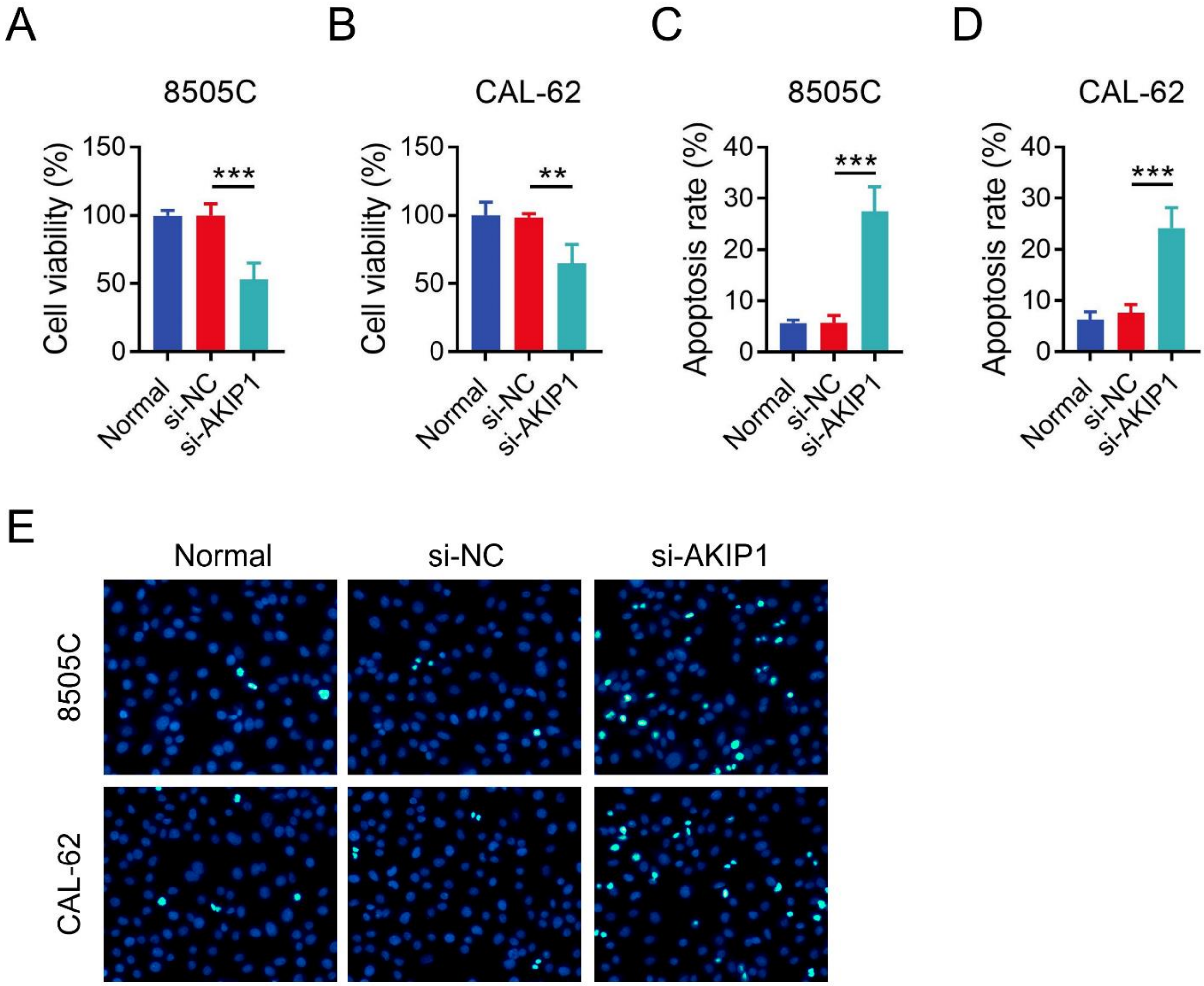

Supplementary figure 1. Cellular functions in ATC cell lines at 72h after siRNA transfection. Comparison of relative cell viability among groups in 8505C cells (**A**) and CAL-62 cells (**B**) after transfection; comparison of apoptosis among groups in 8505C cells (**C**) and CAL-62 cells (**D**) after transfection by one-way ANOVA followed by Dunnett's post hoc test; example image of cell apoptosis through TUNEL Apoptosis Assay Kit (**E**) in 8505C cells and CAL-62 cells after transfection. ATC, anaplastic thyroid carcinoma; siRNA, small interfering RNA; AKIP1, A-kinase interacting protein 1; **, *P*<0.01; ***, *P*<0.001.

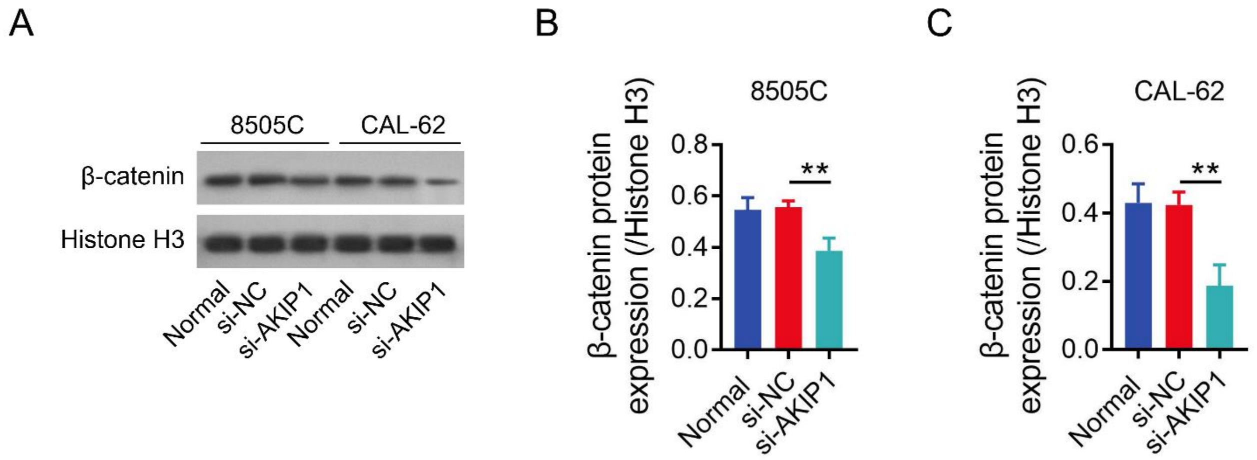

Supplementary figure 2. Nucleus β-catenin expression in ATC cell lines after siRNA transfection. The presentation of western blot images (**A**) and comparison of nucleus β-catenin expression in 8505C cells (**B**) and CAL-62 cells (**C**) after transfection by one-way ANOVA followed by Dunnett's post hoc test. siRNA, small interfering RNA; AKIP1, A-kinase interacting protein 1; **, P<0.01.

A

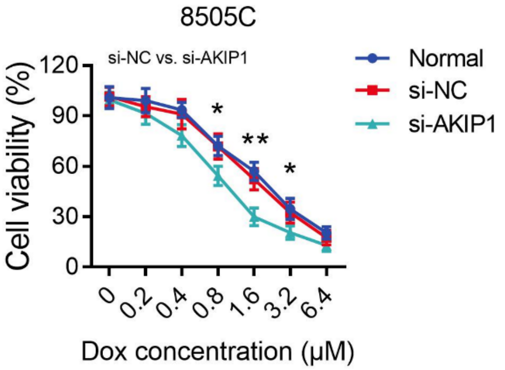

Group	Normal	si-NC	si-AKIP1
IC_{50} (μM)	2.00	1.79	0.97
si-NC vs. si-AKIP1	$p < 0.01$		

B

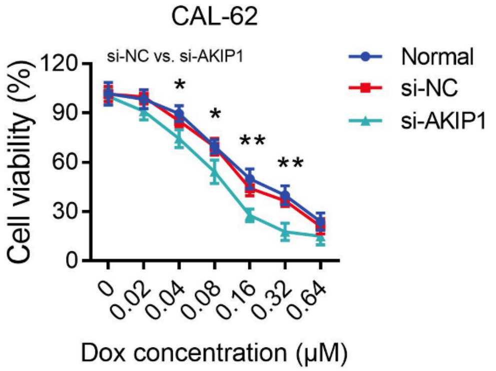

Group	Normal	si-NC	si-AKIP1
IC_{50} (μM)	0.20	0.17	0.09
si-NC vs. si-AKIP1	$p < 0.05$		

Supplementary figure 3. Inhibitory curves of ATC cells after siRNA transfection and doxorubicin treatment. AKIP1 modification regulated cell viability under different concentration of doxorubicin in 8505C cells (**A**) and CAL-62 cells (**B**) by one-way ANOVA followed by Dunnett's post hoc test. AKIP1, A-kinase interacting protein 1; Dox, doxorubicin; IC_{50}, half maximal inhibitory concentration; siRNA, small interfering RNA; NC, negative control *, P<0.05; **, P<0.01

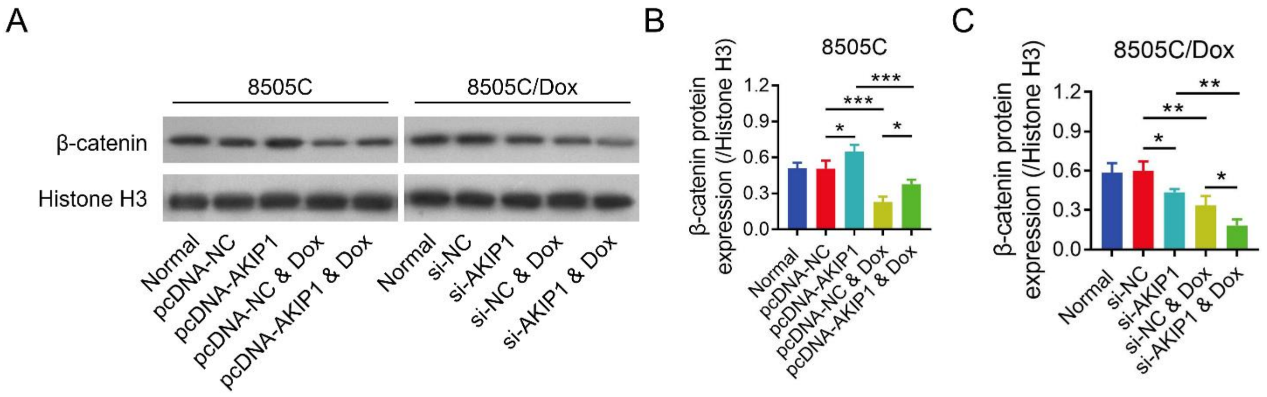

Supplementary figure 4. Nucleus β-catenin expression after AKIP1 modification and doxorubicin treatment in ATC cell lines. The presentation of western blot images (**A**) and comparison of nucleus β-catenin expression in 8505C cells (**B**) and 8505C/Dox cells (**C**) after AKIP1 modification and doxorubicin treatment by one-way ANOVA followed by Tukey's post hoc test. AKIP1, A-kinase interacting protein 1; Dox, doxorubicin; *, $P<0.05$; **, $P<0.01$; ***, $P<0.001$.
